# Shaping Future Directions for Breakdance Teaching

**DOI:** 10.3389/fpsyg.2022.952124

**Published:** 2022-07-05

**Authors:** Ming Tao Wei, Zhi Yang, Yu Jie Bai, Ning Yu, Chun Xia Wang, Nan Wang, Yan Shuo Cui

**Affiliations:** ^1^Teachers College, Changshu Institute of Technology, Suzhou, China; ^2^Melbourne Graduate School of Education, The University of Melbourne, Melbourne, VIC, Australia; ^3^Faculty of Education, Shaanxi Normal University, Xi’an, China; ^4^Department of Dance, Xi'an Conservatory of Music, Xi’an, China

**Keywords:** breakdance, formative assessment, Olympics, rubric/graded scoring key, hip-hop culture

## Abstract

This article reports on the evolution of breakdance. Given the inclusion of breakdancing in the 2024 Olympic Games in Paris, France, scholars have generated substantial international research related to breakdance teaching in recent years. However, few researchers have focused on the impact of formal formative assessment on breakdance teachers’ teaching and students’ learning. We wish to contribute to the quality of breakdance teaching and learning by identifying the positive impact of recent research on formative assessment on student learning and designing a formal formative assessment task related to breakdance. This article lays out a framework of formal formative assessment tasks and identifies the positive impact of formative assessment on dance education. Although our work is far from perfect, it does provide a general methodological framework for assessing breakdance students’ abilities in formal educational settings.

## Introduction

Breaking is the oldest known hip-hop style of dance. It is believed to have originated in the Bronx, New York, in the 1970s (Yang et al., 2022). In the following sections of this article, we thoughtfully chose the term “breakdancing” in order to make this art form easily and clearly conveyed to the general public. We believe this is a reasonable step to promote the culture of “breaking.”

As a key element of hip-hop culture, breakdancing has triggered a domino effect (Li and Vexler, 2019). This effect has propelled it to spread widely around the world. According to research, more than one million people in France participate in breakdance, with around 11 international breakdancing events and 560 national breakdance events each year (Shapiro, 2004). In China, breakdance is one type of street dance. In 2013, China Hip Hop Union Committee (CHUC) was founded by the Chinese Dancers Association, which allowed the art form to be recognized by the professional dance system and gained support from the government (CHUC, 2020). According to CHUC (2020) data, 32 street dance alliances have been founded in China’s 32 provinces (autonomous region, municipality, and special administrative region), with about 8000 street dance institutions in alliances, over three million street dance employees, and over 10 million street dance lovers nationwide. Notably, in 2019, the annual value of production of China’s street dance-related industries has reached RMB 50 billion (7.5 billion USD; CHUC, 2020).

Breakdance is so attractive that even the Olympics wish to include it in their list of events. In 2020, breakdancing became an official Olympic sport. In an effort to attract young people who may not follow some of the traditional sports, the International Olympic Committee (IOC) has officially added the form of street dance to the medal events program for the 2024 Summer Olympics in Paris (IOC, 2021). In addition, breakdance is included in the Olympics which will accelerate its spread around the world. According to research, breakdancing is included in the Olympics which will increase its social attention (Tai et al., 2022), inspire the interest of national governments to support, and promote breakdance (Chang and Lee, 2022), parents will support and encourage their children to learn breakdancing in dance schools (Yang et al., 2022), and new employment opportunities will be created for practitioners (Li and Vexler, 2019). Breakdance is far more beneficial and potent than we might imagine. In China, some universities have introduced breakdancing as an item on their college entrance exams (Li and Vexler, 2019). This certainly gives art students who love breakdancing a new opportunity to pursue higher education.

Given the inclusion of this dance form in the 2024 Olympic Games in Paris, France, scholars subsequently generated a substantial body of international research related to breakdance teaching (Shimizu and Okada, 2018; Vexler, 2020; Gunn, 2021; Dodds, 2022; Tai et al., 2022; Yang et al., 2022), for example, the relationship between deliberate practice theory and breakdance movement proficiency (Shimizu and Okada, 2018) and how the process of learning breakdance reflects dancers’ personal identity (Dodds, 2022). In fact, there is a tendency for researchers to be eager to seek an innovative teaching and learning strategy to help students learn breakdance effectively. Recognizing the importance of improving breakdance teaching methods, in early 2021, we conducted an investigation and discussed what teachers could do to help breakdancing students learn more effectively. The result of that discussion was a call to action that appeared in *Frontiers in Education* (Yang et al., 2022). Again, we wish to contribute to the quality of breakdance teaching and learning by identifying the positive impact of recent research on formative assessment on student learning and designing a formal formative assessment task related to breakdance.

Formative assessment use in dance education has gained increasing importance in making many types of decisions from school improvement to classroom and instructional decision-making. We present this overview based on the following three studies:

- According to Andrade et al. (2015), formative assessments can effectively promote and motivate students to learn dance and even attract students to participate in the classroom.
- According to Overby et al. (2013), formative assessments can help teachers and school administrators identify changes and improvements needed for the future development of the school by observing student responses.
- According to Harding (2012), formative assessments contribute to the reinforcement of dance teachers’ personal values, supporting student leadership roles among their peers, and allowing dance teachers and students to participate in an ever-evolving loop of feedback that is multidirectional.

Such assessment becomes formative assessment when the evidence is actually used to adapt teaching work to meet learning needs (Duckor and Holmberg, 2019b). This means that assessors and teachers must gather evidence about the adequacy of students’ responses to assessment items to help teachers adapt their teaching work. It would be interesting to see if evidence exists that addresses the adequacy of student responses to assessment items in formative assessment articles related to breakdance. The present article attempts to identify gaps in formative assessment tasks related to breakdance and to design what we believe are assessment tasks that effectively identify the competency level of breakdance students. The assessment tasks include assessment items, constructing maps, and judgment-based rubrics.

## Materials and Methods

This article has collected formative assessment tasks related to breakdance from the EBSCO and Web of Science databases. We did not explicitly use the word “breakdance” to search because researchers may focus on related aspects of breakdance such as hip-hop dance (Vasil, 2020), but do not use the word breakdance. However, the articles searched must have included the process of formative assessment task design relevant to breakdance. The following inclusion criteria included (1) written in English, (2) peer-reviewed articles, (3) related to breakdance, and (4) articles (or supplemental materials) must include items or criteria related to assessment skills. To ensure our review was appropriately justified, we used Messick’s six aspects of validity to validate formative assessments related to breakdancing (Messick, 1995). These six aspects include:

- Content relevance (to what extent is the assessment content relevant to the domain of the construct measured).
- Substantive (what is the empirical evidence that the students are actually engaged in the processes that are intended by the assessment).
- Structural (how faithful is the scoring structure of the assessment to the structure of the construct domain measured by the assessment).
- Generalizability (to what extent do score properties, interpretations, and test criterion relationships generalize to and across population groups, settings, and tasks).
- External (to what extent are the assessment items/tasks relevant to the criterion).
- Consequential (what are the actual and potential consequences of using the scores).

## Results

Only two of approximately 25 publications reviewed in the full text were considered to meet the previously stated inclusion criteria (Foley, 2016; Vasil, 2020). Despite early breakdancing educator Foley explicitly calling for students to learn breakdance at the earliest possible age (Foley, 2016), scholarship on formal formative assessments related to breaking has not followed this trend. This phenomenon may hinder teachers from improving the learning of breakdance students. Based on the articles by Foley (2016) and Li and Vexler (2019), we summarize the following three possible reasons:

- For some street styles, historical documents might be difficult to find because they have been lost or were never created in the first place. Thus, this phenomenon may hinder the design of formal formative assessment items, such as match and multiple-choice items.
- Some media companies use breakdancing to spread misinformation for the purpose of advancing their own agendas. The motives for spreading misinformation are many, including self-promotion and attracting attention as part of a business model. This misinformation has led to ambiguous understandings of the concepts of breakdancing, breaking and popular dance, and street dance among the general public. For this reason, we have chosen to use the term “breakdancing” in this article so that it can be more easily searched by the general public and researchers who are not aware of this art form.
- Debate over the status of breakdancing as an Art or Sport continues. At the extremes, some believe breakdancing is a sport. At the other extreme, others think breakdancing is an art. According to the characteristics of an art form and the philosophy of hip-hop culture, no dancer can be called “the best in the world.” Notice that the hip-hop art emphasizes that no dancer can be called the best probably conflicts with summative assessment’s purpose for grading, placement, and classification of students (Dixson and Worrell, 2016). If scholars do not fully understand the blurred relationship between formative assessment, summative assessment, and the characteristics of hip-hop art, then this will hinder the design of assessment tasks.

## Review Assessment Tasks Related to Breakdance

This section will focus specifically on the breakdancing assessment items, criteria, and preparation for students before the assessment. Again, space limitations make an extended discussion of these findings impractical. Interested readers are therefore referred to other relevant sources (Foley, 2016; Vasil, 2020; Sato and Hopper, 2021).

- According to Messick’s six aspects of validity, Vasil’s assessment task was a low-stakes and formative assessment (Vasil, 2020). The results of the assessment were used in a proposal for bringing pop culture into the arts-integrated curriculum. A summative assessment will be conducted at the end of the course, and students will perform a breakdance show. Through three music lessons and three art lessons, students avoid, to some extent, unexpected reactions to the assessment items (Messick, 1995). For example, students will not be confused about the assessment items.
- Vasil’s assessment task also follows, to an extent, the six equity themes proposed by Rasooli et al. (2018).

1. A harmless and constructive classroom environment: Students are explicitly told not to use the second video as learning material (the video has swearing).
2. Transparency, consistency, and justification: Students are provided with courses related to assessment skills.
3. In collaborative activities, students can share their thoughts and ideas and support each other.
4. Avoidance of score pollution: Evidence was obtained through collecting students’ artwork and informal ways (teacher observation). Although no explicit scoring guidelines were provided, the criteria provided in the supplemental materials were sufficient to support teachers’ judgments about student performance.
5. From the pictures provided in Vasil’s article, the space for learning dance may be too narrow. This may not meet the principle that students are given the quality resources needed for assessment.
6. Accommodations: Evidence indicates that students were actively engaged in the assessment process and that no students had unexpected reactions to the assessment.

- Based on the six best recommendations from Mehrens et al. (1998) on preparing students for performance assessments, reviewed below.

1. Determine that the interpretation from student performance is only relevant to the specific task.
2. Teachers did not provide additional guidance to any students prior to the assessment.
3. Students were not confused about the dance performance and drawing items, and teachers provided guidance related to the skills.
4. No evidence that assessment criteria were delivered to students.
5. Students were given small bags filled with leftover materials from art class and were told to create something new (the transferability of assessment skills and knowledge).
6. Students’ artwork was displayed in the hallway for all to view, supporting students to self-assess.

So far, we have reviewed the assessment tasks related to breakdancing. Despite some limitations, especially related to the preparation prior to the assessment, these items are sufficient to be used for the first report of breakdance students’ performance abilities, provided this is not the only assessment that contributes to the report. In addition, we found that existing items on formative and summative assessments are insufficiently designed to cover topics related to breakdancing. When these items are used to assess students, this potentially leads to constructed underrepresentation and affects the reliability of the assessment results (Messick, 1995). In fact, we are certainly not the first to notice this imbalance, but little has been done to address it. We believe that if this article is to be truly helpful to breakdance teachers who continue to face teaching dilemmas, we need to attempt to design a set of formal formative assessment tasks that are directly related to breakdancing. We consider this a reasonable initial step toward a more elaborate intellectual endeavor in the future.

## Assessment Task Design

This section will design a formal formative assessment task related to breakdancing. This is a low-stakes formative assessment task. The assessment is suitable for breakdancing students age eight and up. Breakdancing teachers can use it to assess their students in the following four capabilities: (1) view breakdancing competition videos to analyze dance movement combinations for level features, (2) execute breakdancing movements’ level features, (3) outline breakdancing history level features, and (4) identify breakdancing terms level features.

**Figure 1:**
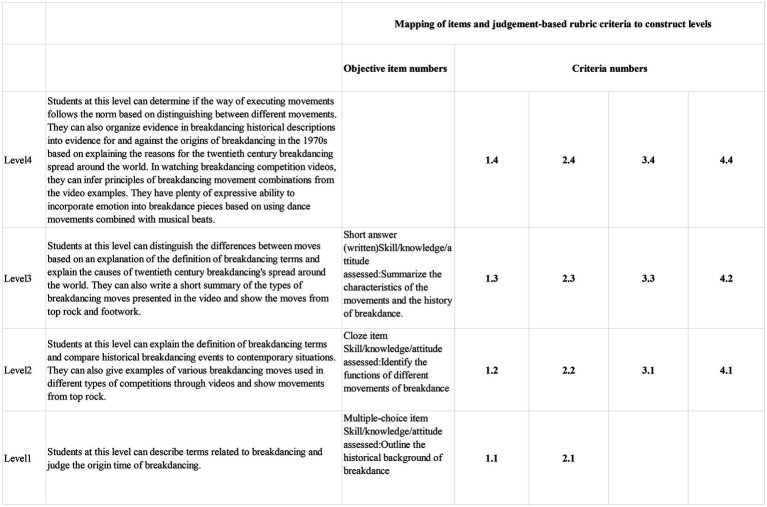
Student Capability Progress Level Map.

The results of the assessment will inform the following decisions: (1) assist breakdancing teachers in improving their teaching strategies, (2) identify breakdancing students’ ability levels and provide formative feedback, (3) breakdancing teachers can use the assessment results to determine students’ position on the construct map and provide targeted guidance to students (See Figure 1; Wilson, 2009; Duckor and Holmberg, 2019a), and (4) the results of the assessment will be discussed at meetings with parents to inform student status.

Some potential constraints on the use of this assessment include: (1) We cannot completely eliminate construct-irrelevant variances during the assessment process because there is always some randomness. As noted by Haladyna and Downing (2004), “we never know the size of random error for any examinee” (p.18). For example, some students may be in a bad mood or distracted during the test, which leads to lost scores on some items, but this does not mean that the student has not mastered the skills assessed. Another example is that some students do not get scores on some items that are too easy, but getting scores on some items that are too difficult, does not mean that the student has mastered the high-level skills as well. Although there may be some randomness during the assessment process, we need to do our best to minimize irrelevant construct variance. (2) Recommendations for minimizing construct-irrelevant variance include the following: (i) the implementation of the assessment needs to follow the six equity themes mentioned in the previous section (Rasooli et al., 2018) and the best recommendations for preparation before the assessment (Mehrens et al., 1998), (ii) a spacious dance classroom, (iii) ensuring the assessment equipment is not damaged (sound, VCR, mirror, and lighting), and (iv) teachers should also provide appropriate protective gear when students are breakdancing.

Ways to record assessment results include: (1) Students’ test scores will be recorded in a notebook by the breakdancing teacher. (2) Scoring guides are provided for each construct item (short answer item and cloze). (3) The recorded scores will be added to an Excel sheet for technical review. (4) At the end of the assessment, teachers should store student’s sample responses effectively to facilitate feedback to students and to support students to self-assess.

The results in the data will be interpreted as follows: (1) As a formative assessment, one of its purposes is to help teachers identify students’ zone of proximal development (ZPD) during teaching and learning and to provide targeted guidance to students. As defined by Vygotsky and Cole (1978), the child’s zone of proximal development (ZPD) is “the distance between the actual developmental level as determined by independent problem solving and the level of potential development as determined under adult guidance or in collaboration with more capable peers” (p.86). For example, some breakdancing students may have excellent performance skills, but they may not be aware of the history of breakdance, so teachers can use the assessment results to target delivery to these students with knowledge related to breakdance. (2) Breakdancing gets Olympic status to debut at Paris Games in 2024. When breakdancing studio teachers consider choosing who would be better able to represent their studio in the dance competition, they can also use the results of the assessment to determine who would be a better choice.

### Item Description

Due to the limited space of the article, it is impractical to describe all items. Thus, we chose short-answer items, multiple-choice, and cloze items as examples to describe. Items types are diverse and to some extent avoid constructing under-representation (Messick, 1994, 1995). A multiple-choice item is designed to assess the learner’s ability to identify the origin and beginning of breakdancing. Multiple-choice items are designed according to the following principles proposed by Haladyna et al. (2002): (1) avoid trick items, (2) simple vocabulary, (3) format vertically rather than horizontally, (4) put the central idea in the stem, (5) avoid negative language, (6) logical option distribution, (7) consistent grammar and structure, and (8) avoid the “none of the above” option.

Cloze items are designed to assess whether the student adequately understands the role of each movement in breakdancing. The design of the fill-in-the-blank items was inspired by Taylor (1956) study that “subjects are asked to close up the gaps in the passages by guessing the identities of the missing words and writing their guesses in the corresponding blanks” (p.43).

The short answer item assesses whether students understand the principles of hip-hop. It requires students to construct their own responses (Rademakers et al., 2005). Responses are presented in multiple words and phrases. The short answer is considered an important item to improve the reliability of the assessment because it can better distinguish between well-prepared students and marginalized students (Fenderson et al., 1997).

In addition, to avoid irrelevant construct variance impacting the reliability of the assessment results, we have done our best to minimize irrelevant construct variance. These efforts include: (1) We collected numerous peer-reviewed articles related to breakdancing to determine the origin of breakdancing. For example, some scholars believe that the origin of breakdancing can be dated back to the 1970s (Forman, 2002; Osumare, 2002; Foley, 2016; Langnes and Fasting, 2016), while others believe that the origin of breakdancing can be traced back much earlier to the 1960s (Bode Bakker and Nuijten, 2018). Due to the controversial origin of breakdancing, the option format was designed as time intervals. (3) For clarity and readability, each option is of a similar length. (4) Providing judgment-based assessment rubric (see Table 1). According to the rules of rubrics proposed by Wolf and Stevens (2007) and Brookhart (2018), the assessment rubric will be reviewed in three iterations.

- First iteration: Ensures that rubrics reflect the quality of the breakdancing student’s cognitive and movement learning, with indicators ordered in terms of increasing proficiency, and using clear and easily understood language avoids ambiguous language.
- Second iteration: Be written so that students can verify their own performance and ensure that defined by a set of quality indicators that display a continuous level of growth.
- Third iteration: Ensure that the criteria match the construct map to help breakdancing teachers identify student position in the construct map (see Figure 1).

**Table 1:** Judgment-based assessment rubric.

1. Identify breakdance movement terms (Indicative behavior criteria):
1.0. Insufficient evidence
1.1. Describe breakdance movement terms
1.2. Explain the definition of breakdance movement terms
1.3. Distinguish the differences between movements based on explaining the definitions of breakdance movement terms
1.4. Determine if the way of execution of movements follows the norms based on distinguishing the differences between movements
2. Construct meaning from breakdance history, including oral and written communication (Indicative behavior criteria):
2.0. Insufficient evidence
2.1. Judging the origin time of breakdance
2.2. Compare historical breakdance events to contemporary situations
2.3. Explain the causes of the twentieth-century breakdance spread around the world
2.4. Organizing evidence in breakdance historical descriptions into evidence for and against the origin of breakdancing in the 1970s, based on explaining the causes of the twentieth-century breakdance’s spread around the world
3. Provide personal insights by watching breakdance videos (Indicative behavior criteria):
3.0. Insufficient evidence
3.1. Give examples of various breakdance movements used in different types of competitions by watching the video
3.2. Classify observed or described cases of breakdance works
3.3. Write a short summary of the types of breakdance moves presented in a video
3.4. Infer principles of breakdance movement combinations from the video examples
4. Showing breakdance movements in the dance studio (Indicative behavior criteria):
4.0. Insufficient evidence
4.1. Shows the top rock movements in breakdance
4.2. Using top rock movements in breakdance combined with footwork movements
4.3. Following the beat of the music based on using a combination of the top rock and transition movements in breakdance
4.4. Incorporate emotions into breakdance pieces based on using dance movements combined with musical beats

Although this is a low-stakes formative assessment task, this process will help design a high-stakes formative assessment task in the future. As mentioned in the introduction, breakdancing has been included in the entrance test program.

#### Multiple-Choice Item

Which of the following is the original date of breakdancing? (Choose an option).

A: Breakdancing originated in the United States between the 1940s and 1950s.

B: Breakdancing originated in the United States between the 1960s and 1970s.

C: Breakdancing originated in the United States between the 1980s and 1990s.

D: Breakdancing originated in the United States between the 2000s and 2010s.

Correct response: **B.**

#### Cloze (Constructed Response)

Breakdancers have many standing steps, such as those known as two-step and Indian-step, which can be categorized as________ (1). In breakdancing, the role of ______ (2) is to connect other dance elements together. ________ (3) refers to movements in which dancers use their ________ (4) and feet to support themselves on the floor; it can also be referred to as shuffle. ________ (5) is a category of street dance, it was announced by the International Olympic Committee to be included in the 2024 Olympic Games in Paris, France.

##### Scoring Guide

Top rock.
Transition.
Footwork.
Hands.
Breaking/b-boying/breakdance/breakdancing.

Words are considered correct if the spelling is close enough to allow the assessor to recognize the words.

#### Short Answer (Written)

What are the five principles of hip-hop?

Sample correct response:

Peace, unity, love, having fun, and knowledge.

##### Scoring Guide

1 point for acknowledging that peace and love are principles of hip-hop.

1 point for acknowledging that unity, having fun, and knowledge as hip-hop principles.

## Conclusion

This article describes the current state of breakdancing. In an effort to attract young people who may not follow some of the traditional sports, the International Olympic Committee (IOC) has officially added the form of street dance to the medal events program for the 2024 Summer Olympics in Paris. In the field of teaching assessment, although early breakdancing educator Foley has explicitly called for students to learn breakdance at the earliest possible age, scholarship on formal formative assessments related to breakdance has not followed this trend. This phenomenon may hinder teachers from improving the learning of breakdance students. We summarized possible reasons for this phenomenon to occur. These reasons include: (i) For some street styles, historical documents might be difficult to find because they have been lost, (ii) some media companies use breakdance to spread misinformation for the purpose of advancing their own agendas, and (iii) debate over the status of breakdancing as an Art or Sport continues. One interesting find is that hip-hop art emphasizes that “no dancer can be called the best,” which probably conflicts with summative assessment’s purpose for grading, placement, and classification of students. Thus, this article claims that if teachers do not sufficiently understand the characteristics of hip-hop culture, then this phenomenon will hinder the design of assessment tasks. Finally, we believe that if this article is to be truly helpful to breakdance teachers who are facing teaching dilemmas, then we need to try to design a formative assessment task. This task is only tentative. We hope that this task will open more research and discussion on formal formative assessment items related to breakdance among scholars in different disciplines. With future research, scholars can help breakdance teachers become more aware of their teaching strategies, which may depend on how teachers perceive students’ test performance.

## Author Contributions

CW collected and organized the literature. NW reviewed the assessment criteria. All authors contributed to the article and approved the submitted version.

## Conflict of Interest

The authors declare that the research was conducted in the absence of any commercial or financial relationships that could be construed as a potential conflict of interest.

## Author’s Note

MW is an associate professor with decades of experience in guiding postgraduate student teams on topics related to international sports phenomena. NY is an associate professor with decades of experience in guiding postgraduate students on topics of teaching methods and educational assessment. ZY is a postgraduate with over 15 years of breakdancing experience and over 10 years of experience as a teacher. YB is a postgraduate with 10 years of dance experience and 5 years of English translation experience. YC is a postgraduate in dance, focusing on the effects of dance therapy on young people's well-being. CW is a postgraduate in education, focusing on creativity in arts education, and in this article. NW is a postgraduate in education and her research interests focus on data literacy and science literacy.

## Publisher’s Note

All claims expressed in this article are solely those of the authors and do not necessarily represent those of their affiliated organizations, or those of the publisher, the editors and the reviewers. Any product that may be evaluated in this article, or claim that may be made by its manufacturer, is not guaranteed or endorsed by the publisher.
